# Epidemiology, Complications, and Surgical Outcomes of Calcaneal Fractures: A Retrospective Single‐Center Study in 202 Patients

**DOI:** 10.1155/aort/5563438

**Published:** 2026-06-30

**Authors:** Thomas Colding-Rasmussen, Müjgan Yilmaz, Anders Wallin Paulsen, Marianne Lind, Michael Mørk Petersen

**Affiliations:** ^1^ Department of Orthopedic Surgery, Rigshospitalet, Copenhagen University Hospital, Blegdamsvej 9, Copenhagen, 2100, Denmark, gentoftehospital.dk; ^2^ Department of Orthopedic Surgery, Hvidovre University Hospital, Kettegaard Alle 30, Hvidovre, 2650, Denmark, hvidovrehospital.dk

## Abstract

**Background:**

Calcaneal fractures are complex injuries associated with a high risk of postoperative complications, including infections and wound healing difficulties. Surgical treatment focuses on restoring anatomical alignment and joint congruency, but these fractures remain challenging to manage. The purpose of this study was to assess the epidemiology, postoperative complications, and surgical outcomes, specifically by evaluating changes in Böhler and Gissane angles in patients with calcaneal fractures.

**Materials and Methods:**

This is a retrospective single‐center cohort study that included 202 patients with 221 calcaneal fractures, 19 of whom had bilateral fractures, treated at Rigshospitalet, Denmark, between 2012 and 2016. Patients were evaluated at 8, 26, and 52 weeks postoperatively. Böhler and Gissane angles were measured preoperatively and postoperatively. Epidemiological data, postoperative complications, and multitrauma cases were recorded.

**Results:**

Of the 221 fractures included, 217 were treated surgically. Among the surgically treated cases, 83% were intra‐articular (*n* = 181) and 17% were extra‐articular (*n* = 36). Twelve patients (6%) had open fractures, and 58 patients (27%) sustained additional trauma‐related injuries, with five classified as multitrauma cases. Preoperative Böhler angles averaged 9.4° (range: −20°–40°), improving to 27.7° postoperatively (range: 10°–45°) (*p* < 0.001). The mean preoperative Gissane angle was 107.4° (range: 52°–147°), improving to 124.1° (range: 102°–147°) postoperatively (*p* < 0.001).

**Conclusion:**

This single‐center study demonstrated significant improvements in Böhler and Gissane angles following surgical intervention for calcaneal fractures. However, postoperative complications, particularly related to soft‐tissue management, remain a significant challenge.

## 1. Introduction

Calcaneal fractures, particularly intra‐articular fractures, are complex injuries that significantly impact patients’ quality of life, often leading to chronic pain, disability, and posttraumatic arthrosis [1]. Surgical intervention aims to restore normal anatomy and joint congruency but is associated with a high risk of complications, such as infection and impaired wound healing [2–4]. Conversely, conservative treatment carries the risk of hindfoot deformity, secondary arthrodesis, and the need for later reconstructive surgery [5, 6]. Initial diagnosis of calcaneal fractures relies on radiographic evaluation, typically supplemented by computed tomography (CT) to better assess fracture lines, articular surface involvement, and subtalar joint congruency, which can be difficult to visualize on conventional X‐rays. CT also plays a critical role in surgical decision‐making and preoperative planning [7].

The Böhler and Gissane angles are important radiological measurements used to assess the severity of calcaneal fractures and guide surgical planning. The Böhler angle, measured on lateral X‐rays, provides an indication of the compression and collapse of the calcaneus, while the Gissane angle assesses the integrity of the posterior facet of the subtalar joint. Both angles are important markers of fracture reduction quality and postoperative outcomes [8].

The optimal treatment for intra‐articular calcaneal fractures remains controversial [9–11]. It is debated whether surgical management leads to superior outcomes compared to conservative treatment, particularly in terms of functional recovery, pain relief, return to work, and prevention of posttraumatic arthrosis [12]. Despite advancements in surgical techniques, reported wound complication rates remain considerable and are highly dependent on the surgical approach [13–15].

Accordingly, there is a need for further evaluation of calcaneus fractures in relation to surgical outcomes, particularly regarding radiological parameters and complication rates. This study evaluates fracture epidemiology, radiological outcomes, including Böhler and Gissane angles, and surgical complications in 202 patients with calcaneal fractures treated at a single orthopedic trauma center in East Denmark between 2012 and 2016.

## 2. Materials and Methods

### 2.1. Study Population

**FIGURE 1:**
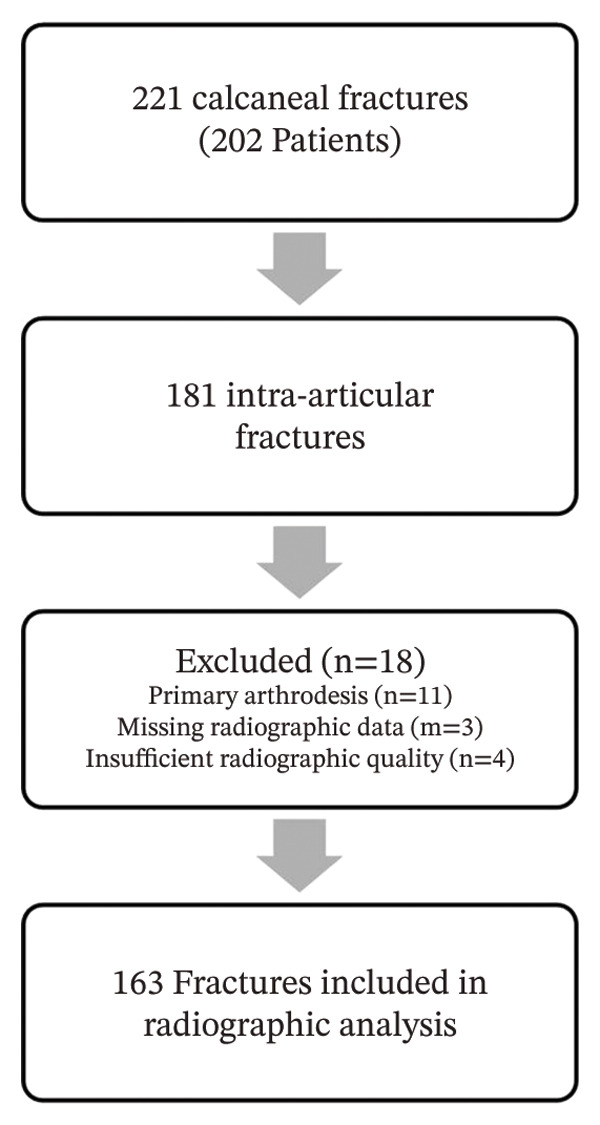
Flowchart illustrating the inclusion and exclusion of calcaneal fractures for radiographic analysis. Of 221 calcaneal fractures identified in 202 patients, 181 were intra‐articular fractures. Eighteen fractures were excluded due to primary arthrodesis (*n* = 11), missing radiographic data (*n* = 3), or insufficient radiographic quality (*n* = 4), resulting in 163 fractures included in the radiographic analysis.

This retrospective single‐center cohort study included 202 consecutive patients with 221 calcaneal fractures, 19 of whom had bilateral fractures, treated due to trauma between 2012 and 2016 (Figure 1). All surgeries were performed at Rigshospitalet, Denmark, by three experienced orthopedic trauma surgeons. In Denmark, calcaneal fracture surgeries are centralized across five centers to ensure adequate surgical expertise and case volume for such fractures [16]. Patients were identified through surgical records and medical coding. Data were collected retrospectively from medical records at postoperative follow‐ups conducted at 8, 26, and 52 weeks. The evaluations at all follow‐ups included assessments of pain levels, medication use, and incidence of revision surgeries. Soft‐tissue complications were specifically assessed at 26 weeks. Variables such as age, gender, smoking status, trauma mechanism, fracture type, surgical details, and postoperative treatment were recorded.

This study was conducted and reported in accordance with the STROBE guidelines. The study was designed as a descriptive and exploratory observational analysis. The primary outcomes of interest were the changes in Böhler and Gissane angles from preoperative to postoperative radiographs. Secondary outcomes included epidemiological characteristics and postoperative complications. No adjustments for multiple comparisons were performed.

### 2.2. Surgical Approach

**FIGURE 2:**
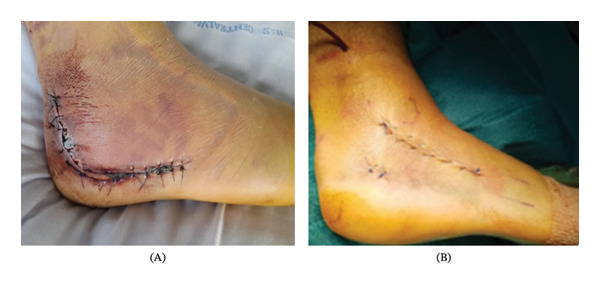
Illustrative patient examples of the two incision techniques used in this retrospective study evaluating 221 calcaneal fractures at a single institution in East Denmark (2012–2016). (A) J/L‐shaped approach and (B) Sinus tarsi approach.

Initially, in the inclusion period, intra‐articular fractures were treated using a J/L‐shaped approach (Figure 2A), but during the study period, there was a gradual shift to the minimally invasive sinus tarsi approach (Figure 2B). The J/L‐shaped approach involves a vascular flap, with the longitudinal incision between the fibula and Achilles tendon, and the vertical incision extending from the base of the fifth metatarsal. The flap is retracted with K‐wires to expose the lateral wall of the calcaneus and, with further dissection, the subtalar joint. After fracture reduction and fixation, the flap is closed in two layers over a drain. With the sinus tarsi approach, an incision is made from the lateral malleolus toward the base of the fourth metatarsal. The sural nerve and peroneal tendons are identified and protected. The sinus tarsi and posterior facet of the subtalar joint are exposed, allowing fracture reduction and fixation with a low‐profile plate. The incision is closed in two layers over a drain. The choice of surgical approach was based on fracture pattern, soft‐tissue condition, and surgeon preference. Over the study period, there was a gradual shift toward increased use of the sinus tarsi approach in selected cases. Timing of surgery was primarily determined by soft‐tissue conditions, including resolution of swelling and appearance of skin wrinkling, as well as logistical factors such as availability of specialized surgeons. Postoperatively, patients followed a standardized departmental protocol with no weight‐bearing for 8 weeks. Range of motion was allowed as tolerated depending on pain and swelling. No systematic differences in postoperative care were applied between surgical approaches.

### 2.3. Böhler and Gissane Angles

**FIGURE 3:**
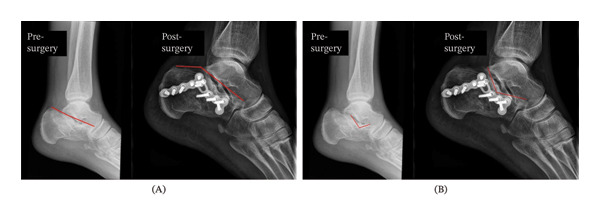
Illustrative patient examples of pre‐ and postsurgery lateral X‐rays from this retrospective study evaluating 221 calcaneal fractures at a single institution in East Denmark (2012–2016). (A) Preoperative (‘pre’) and postoperative (‘post’) Böhler angles, and (B) Preoperative (‘pre’) and postoperative (‘post’) Gissane angles, demonstrating the evaluation of changes following surgical intervention.

Two experienced orthopedic surgeons independently measured the pre‐ and postoperative Böhler and Gissane angles on lateral X‐rays, blinded to each other’s results. No inter‐ or intraobserver reliability analysis was performed, as this descriptive study was not designed to assess measurement reliability. No systematic postoperative CT‐based assessment of reduction quality was performed in this study. The mean value of their measurements was reported for all patients. The Böhler angle is the angle formed by the intersection of a line drawn from the cephalic part of the calcaneal tuberosity to the highest point of the posterior facet and a line from the highest point of the posterior facet to the highest point of the cuboid articular surface (Figure 3A). Normal values range between 20 and 40 degrees [17]. The Gissane angle is formed by the intersection of a line drawn from the posterior talar articular surface through the sinus tarsi and another line from the sinus tarsi to the highest point of the cuboid articular surface (Figure 3B). Normal values range between 130 and 145 degrees [18].

### 2.4. Statistical Analysis and Ethical Approval

Descriptive statistics, including mean and range, were used to describe the study population. Pre‐ and postoperative Böhler and Gissane angles were compared using a paired *t*‐test. Statistical significance was defined as *p* < 0.05, with 95% confidence intervals (95CI). All statistical analyses were performed using RStudio® (Version 1.2.1335). This study was conducted in accordance with the principles of the Declaration of Helsinki. Approval for the study was obtained from the local Ethical Committee (case no. 3‐3013‐2289) and the Danish Data Protection Agency (case no. RH‐2017‐305 and I‐Suite nr: 05913).

## 3. Results

### 3.1. Fracture Characteristics

A total of 221 calcaneal fractures were recorded during the study period, with 19 patients having bilateral fractures. The mean age of the study population was 49.6 years (range 7–91), with 74% males (*n* = 149) and 26% females (*n* = 52). Smoking was reported in 34% (*n* = 68), and diabetes mellitus in 2% (*n* = 4). The most common trauma mechanism was fall from height (81%), followed by traffic accidents (9%) and miscellaneous causes including sports injuries and other accidents (10%).

Two hundred and seventeen cases (98%) were treated surgically. Among these, intra‐articular fractures accounted for 84% (*n* = 181), while extra‐articular fractures comprised 16% (*n* = 36), Figure 1. Among the surgically treated intra‐articular fractures, 95 patients (52%) underwent surgery using the J/L‐shaped approach, while 86 patients (48%) were treated using the sinus tarsi approach. The mean time to surgery was 8.3 days (range: 0–31 days), and the mean duration of surgery was 105 min (range: 30–210 min). Twelve patients (6%) presented with open fractures, 42 patients (20%) sustained injuries while at work, and 15 patients (7%) were foreign citizens. Fifty‐eight patients (27%) had additional injuries related to the trauma, including injuries to the upper extremity (*n* = 12), lower extremity (*n* = 22), internal organs (*n* = 9), spine (*n* = 16), head (*n* = 2), and pelvis (*n* = 2). Among these, five patients were classified as having multitrauma.

### 3.2. Böhler and Gissane Angles

**FIGURE 4:**
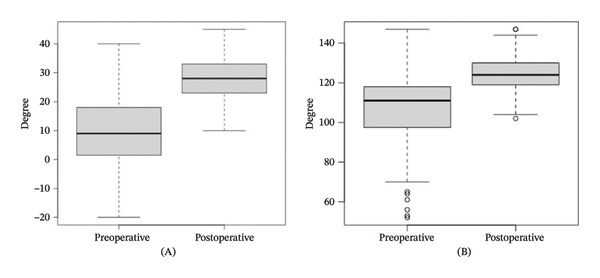
Pre‐ and postoperative Böhler angles (A) and pre‐ and postoperative Gissane angles (B) shown in box plots, from this retrospective study evaluating 221 calcaneal fractures at a single institution in East Denmark (2012–2016).

Of the 181 intra‐articular fractures, 163 were included in the radiographic analysis. Eleven fractures treated with primary subtalar arthrodesis at the time of initial surgery, and three cases with missing radiographs were excluded, and an additional four fractures were excluded due to insufficient image quality for reliable angle measurement. Three further patients underwent secondary subtalar arthrodesis within 52 weeks as part of revision surgery. The mean preoperative Böhler angle was 9.4 degrees (range: −20–40). Postoperatively, the mean Böhler angle increased to 27.7 degrees (range: 10–45), with a statistically significant difference between preoperative and postoperative values (*p* < 0.001) (Figure 4A). The mean preoperative Gissane angle was 107.4 degrees (range: 52–147). Postoperatively, the mean Gissane angle increased to 124.1 degrees (range: 102–147), also showing a statistically significant difference (*p* < 0.001) (Figure 4B).

### 3.3. Follow‐Up

Follow‐up was completed in 124 of 202 patients (61%) at 8 weeks, 113 of 202 patients (56%) at 26 weeks, and 85 of 202 patients (42%) at 52 weeks. At the 8‐week follow‐up (*n* = 124), 63 patients (51%) reported severe pain, and 92 (74%) patients reported daily use of analgesics. Wound complications were observed in 21 patients (17%), which included beak fractures (*n* = 5), sustentaculum fractures (*n* = 2), and Sanders Type 2–4 fractures (*n* = 14). Four of these patients had open fractures at the time of injury. The surgical approaches used were sinus tarsi (*n* = 7) and the J/L shape approach (*n* = 9). Seven patients (6%) underwent early secondary surgery for the removal of orthopedic implants within the follow‐up period. By 26 weeks, three patients continued to experience wound complications, resulting in an overall wound complication rate of 2.7%. One patient experienced severe complications, including necrosis of the calcaneus and Achilles tendon, necessitating an amputation; this occurred in an elderly patient with a beak‐type fracture, delayed diagnosis due to an initially missed fracture, diabetes mellitus, and smoking history. At 26 weeks postoperatively, 33 out of 113 (29%) patients of working age with available data had not yet returned to work, and at the 52‐week follow‐up, 25 out of 85 patients (29%) were still unable to resume work. Additionally, by the 52‐week follow‐up, 14 patients had undergone removal of orthopedic implants and 3 patients had undergone arthrodesis.

## 4. Discussion

In this study, 221 calcaneal fractures (202 patients) were evaluated with pre‐ and postoperative Böhler and Gissane angles. We found a mean preoperative Böhler angle of 9.4 degrees, which increased to 27.7 degrees postoperatively. The corresponding values for the Gissane angle were 107.4 and 124.1 degrees, respectively, with statistically significant changes in both angles when comparing pre‐ and postoperative measurements.

These findings align with previous studies that report significant improvements in Böhler and Gissane angles following surgical treatment [19]. Correction of the Böhler angle is associated with improved outcomes, and an initially small angle is considered a prognostic factor for poor results [20–22]. Thus, we believe that measuring Böhler and Gissane angles is crucial in both diagnosis and follow‐up of calcaneal fractures. However, it is important to note that the accuracy of these measurements depends on who performs them. Otero et al. [23] reported poor reliability, even among experienced personnel, in assessing these angles in displaced intra‐articular calcaneal fractures. Similarly, Gonzales et al. [24] found that observer experience and poor lateral radiograph quality were significant factors contributing to errors, with a margin of error of ±6 degrees for Böhler angle measurements.

Calcaneal fractures are often caused by high‐energy trauma and are frequently associated with multiple injuries. In our study, 26.7% of patients had additional injuries beyond the calcaneal fracture. Although direct comparison is difficult, Diacon et al. [25] also reported associations between hindfoot injuries and pelvic, spinal, ipsilateral, and contralateral lower extremity fractures, as well as intracranial injuries. Additionally, Fitschen‐Oestern et al. [26] noted that 6.5% of foot injuries were missed during the initial diagnosis of multitrauma patients, underscoring the importance of thorough evaluation in such cases. These findings highlight the need for orthopedic surgeons to carefully assess for associated injuries and ensure no foot fractures are overlooked in multitrauma patients.

In this study, two surgical approaches were used: the J/L (extended lateral) approach and the sinus tarsi approach. Surgical management of calcaneal fractures varies, with risks of soft‐tissue complications remaining high. Reported rates range from 17.8% to 30% [27–29]. Van der Vliet et al. [29] found a 30% complication rate for open surgical treatment of beak fractures, compared to 12% for closed procedures. In our study, soft‐tissue complications were reported in 16.9% of patients at 8 weeks postoperatively. However, this figure may be underestimated due to follow‐up challenges: Follow‐up was completed in 61% of patients at 8 weeks, 56% at 26 weeks, and 42% at 52 weeks, with loss to follow‐up particularly among foreign workers who returned to their home countries, potentially leaving complications unreported. Despite advances in preoperative care, surgical techniques, and postoperative management, soft‐tissue complications and infections remain significant postoperative challenges. Further improvements in these areas are needed to reduce complication rates.

This study has several limitations. Its retrospective design limits the data available for follow‐up, and lack of compliance introduces potential selection bias. As a result, the true complication rate may be higher than reported, as patients lost to follow‐up—particularly foreign patients returning to their home countries—may have experienced complications that were not captured in our dataset. Furthermore, while Böhler and Gissane angles were measured by two experienced surgeons, no inter‐ or intraobserver reliability analysis was performed, which could have strengthened the study’s scientific validity. Furthermore, no adjusted comparisons between surgical approaches were performed, as this was an exploratory descriptive study and not designed for comparative or multivariable analyses. Moreover, CT‐based reduction metrics were not systematically assessed in this study, as radiographic evaluation was based on conventional lateral radiographs in accordance with the study design, which limits conclusions regarding subtalar joint congruity. Furthermore, this study did not evaluate functional outcomes or analyze wound complications in relation to Böhler and Gissane angles or surgical approaches, as these questions were beyond the scope of this descriptive study. Additionally, this study did not assess the impact of polytrauma status on surgical timing or radiographic outcomes, and no sensitivity analyses were performed to assess the potential impact of missing data. These aspects should be explored in future research. Lastly, complications were identified based on clinical documentation and were not categorized according to predefined severity criteria, which may limit comparability with other studies. The relatively large sample size and the single‐center design are important strengths as it facilitates consistency in surgical technique and postoperative care, which helps reduce variability in treatment protocols.

## 5. Conclusions

This study demonstrates that surgical intervention for calcaneal fractures results in statistically significant improvements in Böhler and Gissane angles. However, soft‐tissue complications remain a major challenge. Furthermore, conclusions regarding functional outcomes are limited due to the absence of patient‐reported outcome measures and detailed clinical outcome data. Further studies are warranted.

## Funding

No funding was received for this research.

## Conflicts of Interest

The authors declare no conflicts of interest.

## Data Availability

The data that support the findings of this study are available from the corresponding author upon reasonable request.
